# Multiparametric magnetic resonance imaging-derived deep learning network to determine ferroptosis-related gene signatures in gliomas

**DOI:** 10.3389/fnins.2022.1082867

**Published:** 2022-12-20

**Authors:** Zhichao Zuo, Wen Liu, Ying Zeng, Xiaohong Fan, Li Li, Jing Chen, Xiao Zhou, Yihong Jiang, Xiuqi Yang, Yujie Feng, Yixin Lu

**Affiliations:** ^1^Department of Radiology, Xiangtan Central Hospital, Xiangtan, Hunan, China; ^2^Department of Radiology, The Third Xiangya Hospital, Central South University, Changsha, Hunan, China; ^3^The School of Mathematics and Computational Science, Xiangtan University, Xiangtan, Hunan, China; ^4^Department of Radiology, Hunan Children’s Hospital, University of South China, Changsha, Hunan, China; ^5^Department of Radiology, The Affiliated Hospital of Southwest Medical University, Luzhou, Sichuan, China; ^6^Medical Imaging Department, Guangxi Medical University Cancer Hospital, Nanning, Guangxi, China

**Keywords:** glioma, ferroptosis, prognosis, MRI, deep learning network

## Abstract

**Introduction:**

Ferroptosis-related gene (FRG) signature is important for assessing novel therapeutic approaches and prognosis in glioma. We trained a deep learning network for determining FRG signatures using multiparametric magnetic resonance imaging (MRI).

**Methods:**

FRGs of patients with glioma were acquired from public databases. FRG-related risk score stratifying prognosis was developed from The Cancer Genome Atlas (TCGA) and validated using the Chinese Glioma Genome Atlas. Multiparametric MRI-derived glioma images and the corresponding genomic information were obtained for 122 cases from TCGA and The Cancer Imaging Archive. The deep learning network was trained using 3D-Resnet, and threefold cross-validation was performed to evaluate the predictive performance.

**Results:**

The FRG-related risk score was associated with poor clinicopathological features and had a high predictive value for glioma prognosis. Based on the FRG-related risk score, patients with glioma were successfully classified into two subgroups (28 and 94 in the high- and low-risk groups, respectively). The deep learning networks TC (enhancing tumor and non-enhancing portion of the tumor core) mask achieved an average cross-validation accuracy of 0.842 and an average AUC of 0.781, while the deep learning networks WT (whole tumor and peritumoral edema) mask achieved an average cross-validation accuracy of 0.825 and an average AUC of 0.781.

**Discussion:**

Our findings indicate that FRG signature is a prognostic indicator of glioma. In addition, we developed a deep learning network that has high classification accuracy in automatically determining FRG signatures, which may be an important step toward the clinical translation of novel therapeutic approaches and prognosis of glioma.

## 1 Introduction

Glioma, the most common primary brain tumor in adults, is highly invasive and resistant to various combination therapies such as surgery, radiotherapy, and chemotherapy (Zhou et al., 2022). In particular, glioblastoma multiforme, which is the most malignant type of central nervous system (CNS) tumor, has a median survival <16 months, and this has not improved substantially with modern medical advances (Ma et al., 2018). Glioma recurrence, progression, and metastasis are the three primary challenges that lead to treatment failure. Previous studies have shown that glioma, as a highly complex and heterogeneous tumor, involves multiple pathways, the immune microenvironment, and metabolic reprogramming during its development and progression (Gao et al., 2022). Recently, molecular markers related to the prognosis and treatment of gliomas have been actively explored. For example, isocitrate dehydrogenase (IDH) and 1P/19q have been confirmed to be associated with the prognosis of glioma. In addition, there are new drugs targeting epidermal growth factor receptor (EGFR) and mammalian target of rapamycin (mTOR) for the treatment of gliomas (Deluche et al., 2019; Heinzen et al., 2019; Hu et al., 2022). Unfortunately, there are still many gaps in the prognostic assessment and treatment of gliomas; therefore, the identification of new markers remains imperative.

Ferroptosis is a form of regulated cell death triggered by lipid peroxidation that differs from other genetic, biochemical, and morphological forms of cell death (Dixon et al., 2012). The mechanism of ferroptosis involves several redox-inducing compounds (e.g., erastin and RSL3) and ferroptosis inhibitors (e.g., ferrostatin-1 and liproxstatin-1). Importantly, some cancer cells that are resistant to compounds targeting traditional cell death processes are susceptible to RSL3- and erastin-induced ferroptosis, indicating that ferroptosis induction may be an encouraging therapeutic strategy for gliomas (Hangauer et al., 2017). Previous studies have indicated that ferroptosis-related gene (FRG) signatures are associated with tumor immune features and have potential for prognosis prediction and immunotherapy assessment in gliomas (Hu et al., 2021; Wan et al., 2021). Thus, it is crucial to accurately predict FRG-related risk to plan an effective curative treatment.

Convolutional neural networks (CNNs) are a form of deep learning widely applied for image processing and cover an extensive range of disciplines, such as molecular profiles and genomic mutations in gliomas (Liu et al., 2021). Even though several hurdles exist for clinical implementation (Chang K. et al., 2018; Chang P. et al., 2018), these image signal intensity-based CNNs did not enable the incorporation of information from the tumor 3D voxel, which may cause data leakage problems. Moreover, the existing methodologies require extensive manual preprocessing, presegmentation, or multicontrast acquisitions, which limit their clinical merits. To mitigate these limitations, in the present study, we developed a fully automated and highly accurate deep learning 3D network and further performed this non-invasive method to determine the FRGs signature.

## 2 Materials and methods

### 2.1 Data collection

mRNA expression data and clinical information in glioma from three public databases (Ceccarelli et al., 2016), The Cancer Genome Atlas (TCGA),^1^ Chinese Glioma Genome Atlas (CGGA),^2^ and Genotype-Tissue Expression (GTEx),^3^ were used in this study. After data filtration, five available datasets, namely CGGA_693, CGGA_325, TCGA_LGG, TCGA_GBM, and GTEx, were chosen for further analyses. Among these datasets, TCGA_LGG and TCGA_GBM were assigned to the training cohort, whereas CGGA-693 and CGGA-325 were assigned to the validation cohort. Differential gene expression (DGE) analysis was conducted based on the GTEx dataset.

Gene transcription levels were normalized as fragments per kilobase million (FPKM) and further transformed to log2 (FPKM+1) for downstream analysis. A batch correction per subclass was applied using R packages “limma” and “sva.” A total of 323 ferroptosis-related genes, including drivers, suppressors, and marker regulators, were obtained from the FerrDb database (Zhou and Bao, 2020)^4^.

### 2.2 Construction of the FRG-related risk score

DGE analysis was conducted using the R package “limma” between tumor and normal tissue samples, and FDR < 0.05 and | logFC| > 1 was set as the threshold. Univariate Cox regression was used for the analysis of independent prognostic factors, and *P* < 0.05 was set as the significance threshold. The intersection of the genes, based on the results of DGE and univariate Cox regression analyses, was mapped using the R package “Venn” and described as FRGs for further analysis.

The defined FRGs were subjected to least absolute shrinkage and selection operator (LASSO) Cox regression, which is a classical dimension-reduction approach to screen for independent prognostic factors. The FRG-related risk score was constructed based on the LASSO weighting coefficients of the final selected genes using the following formula:


$$\sum_{i=1}^{n}\left(Coef_{i}\times x_{i}\right),$$

where Coef_i_ represents the coefficients and x_i_ is the FPKM value of each FRG.

### 2.3 FRG-related risk score stratification

We used the R package “survminer” to classify the FRG-related risk scores into low- and high-risk groups. The survival rate differences among the stratified groups were compared using Kaplan–Meier (KM) analysis along with log-rank tests. Time-dependent receiver operating characteristic (tROC) curves were used to assess the efficiency of the FRG-related risk score in prognostic prediction.

To compare the clinicopathological and molecular characteristics between the low- and high-risk groups, Chi-square or Student’s *t*-tests were used. Statistical significance was set at *P* < 0.05.

### 2.4 Gene ontology and Kyoto encyclopedia of genes and genomes

A functional annotation of differentially expressed genes was used to visualize gene ontology (GO) and Kyoto Encyclopedia of Genes and Genomes (KEGG) results using the R package “ClusterProfiler,” to further explore their functional correlation.

### 2.5 Imaging acquisition and preprocessing

This study used multiparametric magnetic resonance (MR) images obtained from The Cancer Imaging Archive (TCIA) (Bakas et al., 2017).^5^ Preoperative MR images of each patient with glioma were obtained from T1-weighted (T1WI), T2-weighted (T2WI), fluid-attenuated inversion recovery (FLAIR), and T1 contrast-enhanced (T1CE) images.

**FIGURE 1:**
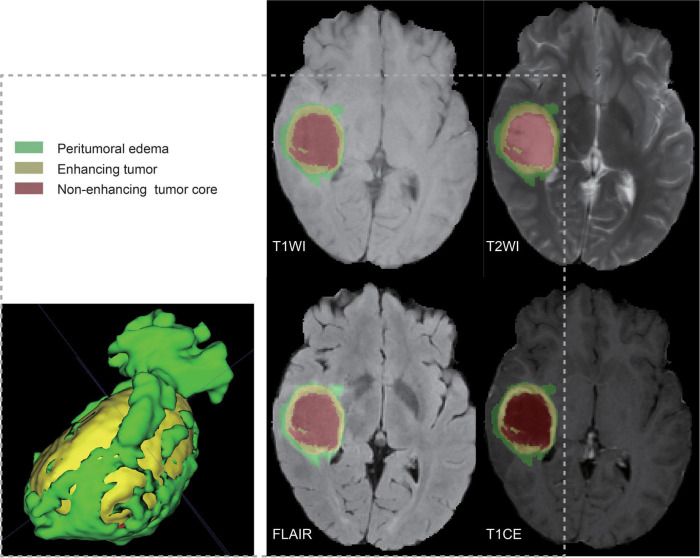
Ground truth whole-tumor masks. Red voxels represent non-enhancing tumor core; yellow voxels, enhancing tumor; and green voxels, peritumoral edema.

A total of 122 patient samples were analyzed, representing all matched cases (according to shared barcodes) of glioma in TCGA and TCIA, of which 28 and 94 were partitioned into the high-risk and low-risk groups, respectively. The multiparametric MR images were preprocessed using the Cancer Imaging Phenomics Toolkit open-source software (CaPTk v.1.9.0) (Pati et al., 2020).^6^ The acquired Digital Imaging and Communications in Medicine (DICOM) images were converted to Neuroimaging Informatics Technology Initiative (NIfTI) images and reoriented to the right-most, anterior-most, inferior-most (RAI) coordinate system. Based on the SRI atlas, it was coregistered and resampled to a spatial resolution of 1 mm × 1 mm × 1 mm (Rohlfing et al., 2010). The anatomical images were bias-corrected and skull-stripped after high-resolution reconstruction. After removing the outlier pixels that did not fall in the 99.9% percentile of the image histogram, the intensities of the images were converted into a background range of 0–255. Automated segmentation was performed using the DeepMedic module (Kamnitsas et al., 2017) and was approved or adjusted when necessary by a board-certified neuroradiologist (Lu) through CaPTk to determine the tumoral subregions of the TC (enhancing tumor and non-enhancing portion of the tumor core) and WT (whole tumor and peritumoral edema) (Figure 1).

### 2.6 Network details

**FIGURE 2:**
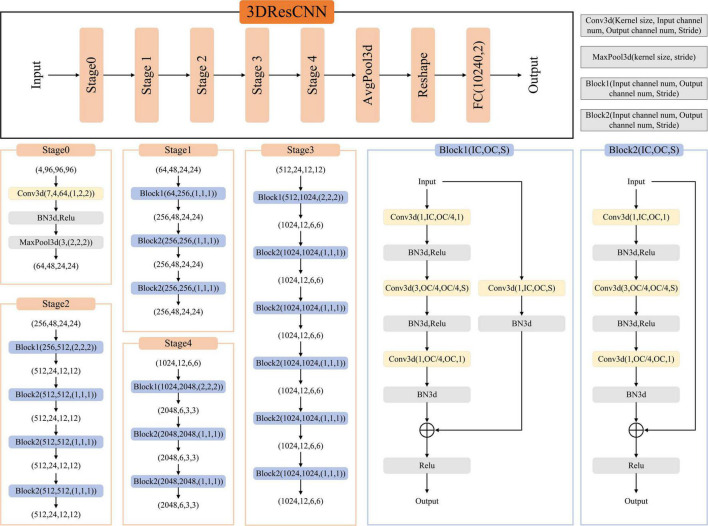
The network framework for FRGs signature prediction.

We used the network architecture shown in Figure 2, a classification network trained on T1WI, T1CE, T2WI, and FLAIR based on TC and WT mask images, respectively. It is a CNN classifier for predicting high- and low-risk FRG-related signatures. The network framework is derived from the classical ResNet50, which contains an initial part (stage0), a residual learning part (stage1–stage4), and a fully connected part (AvgPool3d+Reshape+FC).

The residual learning part (stage1–stage4) comprises three, four, six, and three residual blocks, respectively. The residual blocks employed here are the Conv Block (Block1) and Identity Block (Block2). The Conv Block has inconsistent input and output dimensions, requiring the addition of a 1 × 1 convolution and Batch Normalization (BN) at the location of the shortcut path to stretch the channels and make the dimensions consistent before the summation operation. The Identity Block (Block2) has consistent input and output dimensions, allowing for straightforward addition. The output of the residual learning part was transformed into data dimensions by AvgPool3d and reshape operations and used as the input of the FC.

#### 2.6.1 Network implementation and cross-validation

To ensure the reliability of the network implementation, threefold cross-validation was performed on data from 122 patients (28 high-risk and 94 low-risk groups), and the dataset was randomly divided into threefolds. The data from these threefolds were alternated between the two training sets and one validation set to obtain three prediction models, and the average performance of these three models was calculated. The input images were T1WI, T1CE, T2WI, and FLAIR images cropped by the TC/WT mask to obtain the data with 96 × 96 × 96 pixels. During network training, we performed random flip operations on the input images for data enhancement. The Cross Entropy Loss was chosen as the loss function of the network. The network learning rate was set to 10^–4^, the batch size was 8, and the maximum number of epochs was 70. Our pipeline was written in Pytorch, and all experiments were performed on a workstation with an Intel Xeon CPU E5-2630 and NVIDIA Tesla V100 GPU.

### 2.7 Network assessment

The diagnostic efficiency was assessed by receiver operating characteristic (ROC) using all predictions across folds of the cross-validation, and the area under the curve (AUC), accuracy (ACC), sensitivity, and specificity were calculated. Besides, we used the precision, recall, and F1 score to assess the signature classification performance of the FRGs. The F1 score was calculated by combining the precision and recall.

## 3 Results

### 3.1 Landscape of FRGs

**FIGURE 3:**
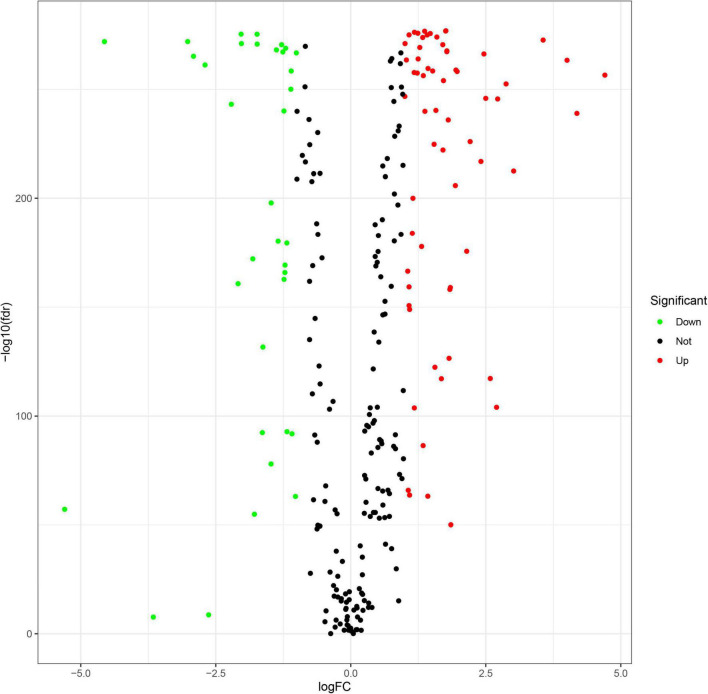
Volcano plot of differential gene expression analysis between tumor and normal tissues.

**FIGURE 4:**
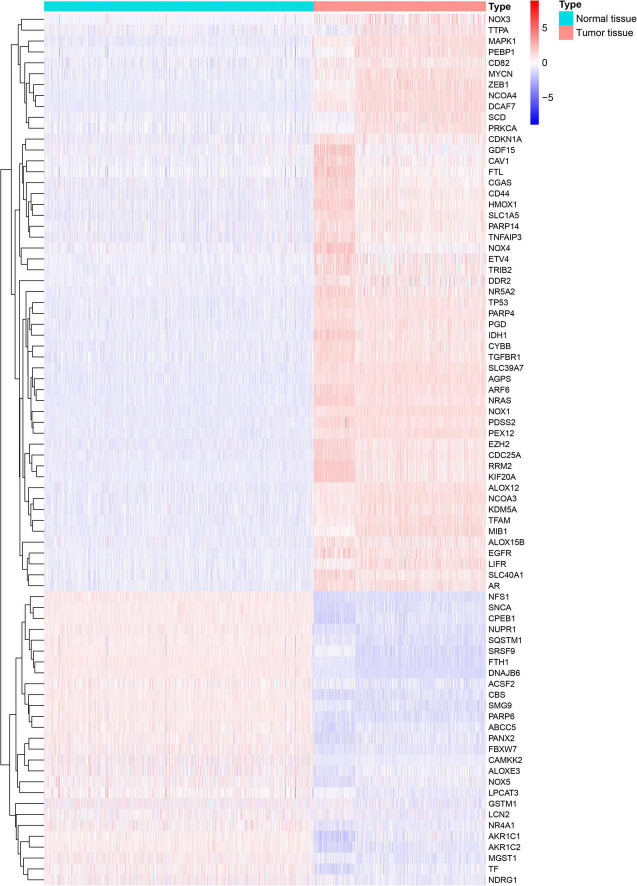
Heatmap generated and displayed as log twofold change.

A flowchart describing our study’s analytical procedure is presented (Supplementary Figure 1). The TCGA cohort containing 270 ferroptosis-related genes was obtained after removing some of the low-expression ferroptosis-related genes. After DGE analysis between tumor and normal tissue samples, 99 genes were retained (Figure 3). In addition, Cox regression analysis identified 221 genes as independent prognostic factors. Taking the intersection of predicted genes, 80 genes described as FRGs, including 53 up-regulated genes and 27 down-regulated genes, were enrolled (Supplementary Figure 2). Upregulation and downregulation of gene expression were also plotted as heatmaps and compared with the corresponding control (Figure 4).

**FIGURE 5:**
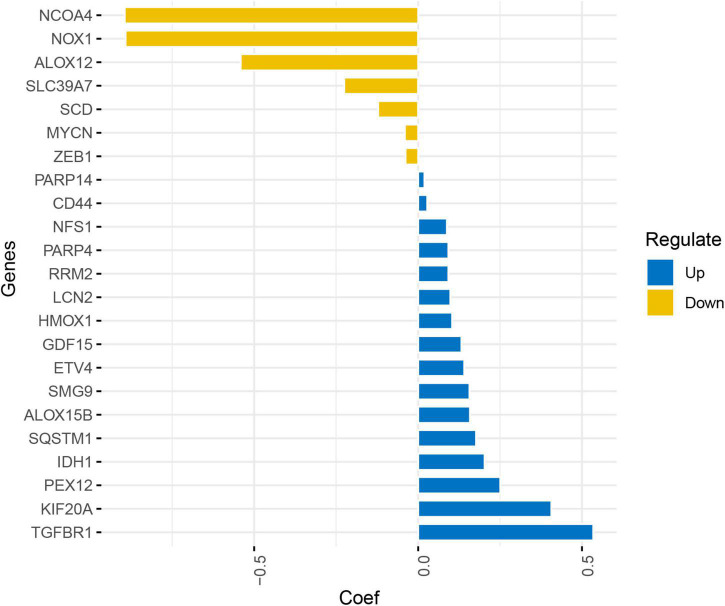
Least absolute shrinkage and selection operator coefficient profile of the candidate genes.

Least absolute shrinkage and selection operator Cox regression analysis was conducted on 80 FRGs and identified 23 genes associated with the prognosis of glioma: 12 driven genes (*NOX1, NCOA4, ALOX12, ALOX15B, ZEB1, HMOX1, TGFBR1, IDH1, PEX12, MYCN, SMG9*, and *SLC39A7*) and 12 suppressor genes (*SCD, NFS1, SQSTM1, CD44, RRM2, GDF15, PARP4, PARP14, KIF20A, ETV4, LCN2*, and *HMOX1*), among which *HMOX1* is both a driver and a suppressor gene. The LASSO coefficient profiles of candidate genes are shown in Figure 5. The FRG-related risk score was calculated according to the LASSO weighting coefficients of the final selected genes.

### 3.2 FRG-related risk score in prognosis

We determined the optimal cut-off using the “surv_cutpoint” function of the “survminer” R package (Supplementary Figure 3). Patients with FRG-related risk scores were further divided into high- and low-risk groups (Supplementary Figure 4).

**FIGURE 6:**
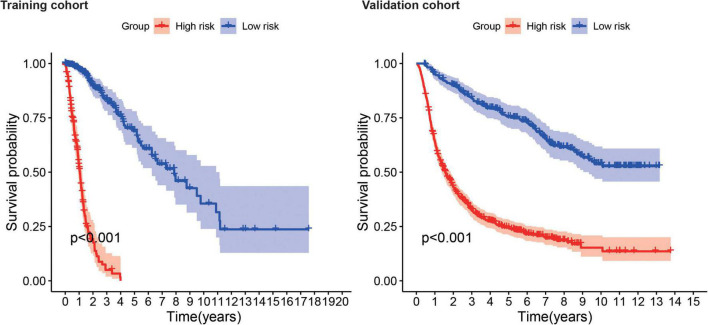
Survival analysis shows survival probability curves in the training and validation cohort.

**FIGURE 7:**
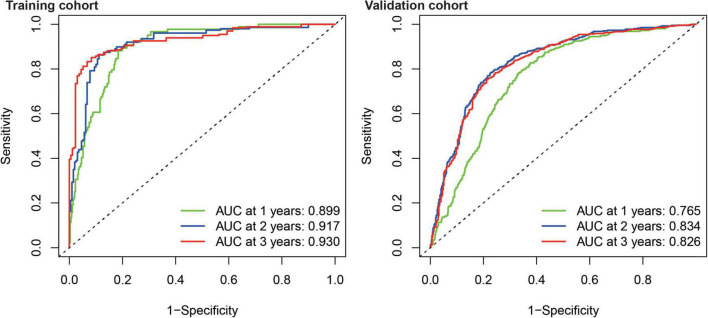
Time-dependent receiver operating characteristic analysis showed the AUC of FRGs for 1, 2, and 3 years in the training and validation cohort.

Kaplan–Meier survival analysis showed that the survival probability was significantly worse in the high-risk group than in the low-risk group in the training and validation cohorts (*P* < 0.001) (Figure 6). tROC curve analysis showed that the development of the risk score in the present study exhibited good predictive effectiveness, which was indicated by 0.899, 0.917, and 0.930 for 1-, 2-, and 3-year AUC, respectively, in the training cohort and 0.765, 0.834, and 0.826 for 1-, 2-, and 3-year AUC, respectively, in the validation cohort (Figure 7).

### 3.3 Association of FRG-related risk score with clinicopathological and molecular characteristics

**FIGURE 8:**
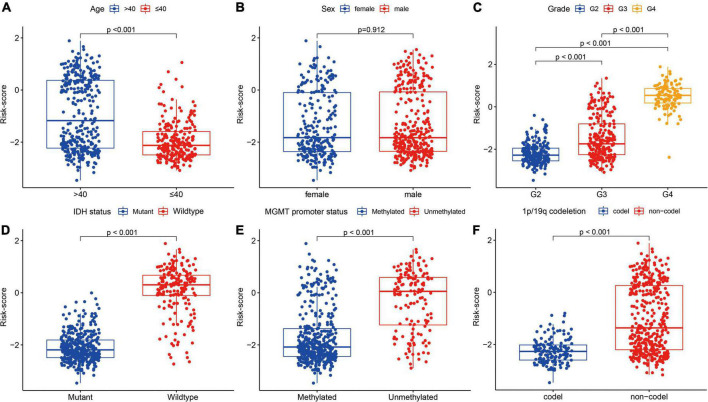
Association between the FRGs and other clinicopathological characteristics (**A**, age; **B**, sex; **C**, grade; **D**, IDH status; **E**, MGMT promoter status; and **F**, 1p/19q codeletion) in the training cohort.

There were significant differences in FRG-related risk scores among patients in age (*P* < 0.001), grade (*P* < 0.001), IDH status (*P* < 0.001), MGMT promoter (*P* < 0.001), and 1p/19q codeletion (*P* < 0.001), but no significant differences were observed in sex (*P* = 0.912) (Figure 8).

In addition, patients in the high-risk group had higher grade, older age, wild-type IDH, 1p/19q non-codeletion, and MGMT promoter unmethylation (*P* < 0.001), but had no significant differences in sex (Table 1).

**TABLE 1:** Characteristics of patients in low- and high-risk groups in the training cohort.

Characteristic	High-risk (*N* = 145)	Low-risk (*N* = 418)	*P*-value
Grade:			<0.001
G2	0 (0.00%)	212 (50.7%)	
G3	40 (27.6%)	196 (46.9%)	
G4	105 (72.4%)	10 (2.39%)	
Age (year)	61.0 (53.0, 70.0)	39.0 (32.0, 51.0)	<0.001
Sex:			0.912
Female	61 (42.1%)	180 (43.1%)	
Male	84 (57.9%)	238 (56.9%)	
IDH status:			<0.001
Mutant	1 (0.69%)	369 (88.3%)	
Wildtype	144 (99.3%)	49 (11.7%)	
1p/19q codeletion:			<0.001
Codel	0 (0.00%)	149 (35.6%)	
Non-codel	145 (100%)	269 (64.4%)	
MGMT promoter:			<0.001
Methylated	63 (43.4%)	356 (85.2%)	
Un-methylated	82 (56.6%)	62 (14.8%)	

### 3.4 Analysis of biological properties and pathways related to the gene signatures

**FIGURE 9:**
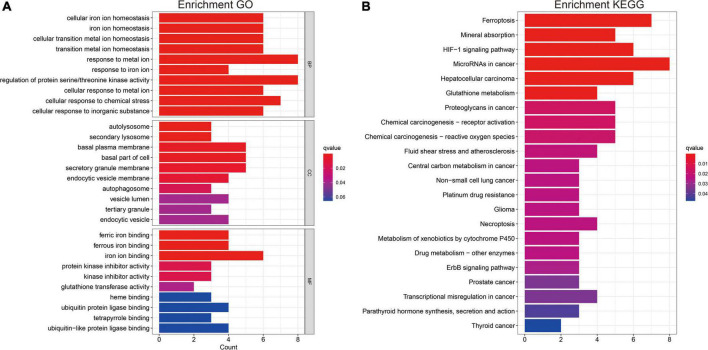
Gene ontology and KEGG analysis **(A,B)**. Functional annotation of FRGs using GO terms and the KEGG pathway.

Gene ontology and KEGG analyses were used to annotate the intersection genes after risk differential analysis, (Figure 9). The biological processes (BPs) involved included the following: Cellular iron ion homeostasis, iron ion homeostasis, cellular transition metal ion homeostasis, transition metal ion homeostasis, response to metal ion, response to iron ion, regulation of protein serine/threonine kinase activity, cellular response to metal ion, cellular response to chemical stress, and cellular response to inorganic substance (Figure 9A). The most abundant cellular component (CC) terminology included autolysosome, secondary lysosome, basal plasma membrane, basal part of cell, secretory granule membrane, and endocytic vesicle membrane (Figure 9A). The most abundant molecule function (MF) terms were ferric iron binding, ferrous iron binding, iron ion binding (Figure 9A). KEGG pathway analysis revealed that ferroptosis, Mineral absorption, HIF-1 signaling pathway, MicroRNAs in cancer, Hepatocellular carcinoma, and Glutathione metabolism were the most abundant pathways (Figure 9B).

### 3.5 Network performance in determining FRG signatures

The cross-validation average ACC of 3DResCNN (network (TC-mask)) reached 0.842 (0.900, 0.850, and 0.775 for Fold1, Fold2, and Fold3, respectively), the average F1 score was 0.843 (0.900, 0.844, and 0.784 for Fold1, Fold2, and Fold3, respectively), and the average AUC was 0.781 (0.827, 0.767, and 0.750 for Fold1, Fold2, and Fold3, respectively).

The cross-validation average ACC of 3DResCNN [network (WT-mask)] reached 0.825 (0.925, 0.800 and 0.750 for Fold1, Fold2, Fold3, respectively), the average F1 score was 0.830 (0.923, 0.810 and 0.757 for Fold1, Fold2, Fold3, respectively), and the average AUC was 0.781 (0.842, 0.800, and 0.700 for Fold1, Fold2, Fold3, respectively). The specific results of 3DResCNN in terms of the threefold respective and average metrics are shown in Table 2.

**TABLE 2:** Network performance in determining FRG signatures.

Metrics	AUC	ACC	F1	Sensitivity	Specificity
**TC-mask**					
Fold1	0.827	0.900	0.900	0.714	0.939
Fold2	0.767	0.850	0.844	0.600	0.933
Fold3	0.750	0.775	0.784	0.700	0.800
**Average**	0.781	0.842	0.843	0.671	0.891
**WT-mask**					
Fold1	0.842	0.925	0.923	0.714	0.970
Fold2	0.800	0.800	0.810	0.800	0.800
Fold3	0.700	0.750	0.757	0.600	0.800
**Average**	0.781	0.825	0.830	0.705	0.857

The network (TC mask) showed a similar performance to the network (WT mask), and the summary ROC curves for the network (TC mask and WT mask) are shown in Supplementary Figure 5.

## 4 Discussion

Evidence suggests that ferroptosis plays a crucial role in tumor initiation, progression, and evolution (Dixon et al., 2012). Several investigations have indicated that the risk score generated by ferroptosis is associated with the clinicopathological features of gliomas, which can independently predict patient prognosis (Zhuo et al., 2020; Hu et al., 2021; Wan et al., 2021). In addition, ferroptosis may affect immune cell infiltration in the glioma microenvironment (Hu et al., 2021; Wan et al., 2021). Bioimaging is an essential tool for the non-invasive diagnosis of gliomas. Indeed, combined with multiple imaging modalities, multiparametric MR imaging enables effective expansion of the feature pool, which would provide more information. Previous studies have indicated that multiparametric MR-based deep learning has diagnostic performance in the differentiation of glioma mimicking encephalitis (Wu et al., 2021), classification of IDH mutation status (Bangalore Yogananda et al., 2020), discrimination of pseudoprogression and true progression (Lee et al., 2020), and determination of molecular subtype in gliomas (Li et al., 2022). For these reasons, we developed a deep learning 3D network and further applied this non-invasive method to assess FRG signatures.

The results of GO and KEGG analysis showed the main enrichment pathways of intersection genes in this study. Considering the genetic diversity, 23 FRGs were finally incorporated into prognostic signatures based on LASSO Cox analysis. Our results indicated that the expression of FRGs was associated with poor clinicopathological features, and the FRG-related risk score had a high predictive value for glioma prognosis, which is consistent with previous literature (Zhuo et al., 2020; Hu et al., 2021; Wan et al., 2021). According to the FRG-related risk score, patients with glioma were successfully classified into high- and low-risk groups, which contributed to prognosis stratification.

To our knowledge, this is the first study to demonstrate the application of multiparametric MRI-derived deep learning network for determining FRG signatures in gliomas. CNNs are the cornerstone of deep learning methods, which remain more efficient in image annotation than classical hand-engineered selections, such as color, geometrical, and texture features (Korfiatis and Erickson, 2019). In the present study, we used 3DRESCNN (ResNet50) to predict FRG-related risk signatures and used multiparametric MRI imaging (T1WI, T1CE, T2WI, and FLAIR images) as network inputs. The network (TC or WT mask) achieved highly satisfactory prediction efficiency, with ACCs of 84.2 and 82.5%, respectively. In reviewing these multiparametric MRI images, there were no specific imaging features, indicating that the deep learning network provided additional information that could not be interpreted manually. The TC mask represented an enhancing tumor and non-enhancing portion of the tumor core, and the WT mask represented the whole tumor and peritumoral edema. WT mask and TC mask demonstrated similar performances, indicating that peritumoral edema did not enhance the diagnostic effectiveness for determining the FRG signatures.

Despite the satisfactory results, the present study has several limitations. First, we developed a multiparametric MRI-derived deep learning network, whereas diffusion-weighted MRI images were not available. Second, training deep learning models usually requires large amounts of data (Choy et al., 2018), whereas the cases for training are limited. Despite the application of data augmentation for CNNs, because of their high complexity and the use of 3DCNN in this study, the amount of data is too small, easily leading to poor network training and performance. Third, owing to the relatively large number of low-risk FRG-related signatures, this type of quantitative imbalance has a significant negative impact on the training of the CNN classifier, which affects both the convergence of the training phase and analysis of the test set results, as well as the threefold validation.

## 5 Conclusion

In conclusion, we developed a multiparametric MRI-derived deep learning network with high accuracy for automatically determining FRG signatures. This study represents an important technological milestone using MR imaging to evaluate genetic diversity, prognosis conditions, and drug-targeted genes for gliomas.

## Data availability statement

Publicly available datasets for this study can be found in the Cancer Genome Atlas (TCGA, https://tcga-data.nci.nih.gov), Chinese Glioma Genome Atlas (CGGA, http://www.cgga.org.cn/), Genotype-Tissue Expression (GTEx, http://xenabrowser.net), FerrDb database (http://www.zhounan.org/ferrdb/current/), and The Cancer Imaging Archive (TCIA, https://wiki.cancerimagingarchive.net).

## Ethics statement

Ethical review and approval was not required for the study on human participants in accordance with the local legislation and institutional requirements. Written informed consent for participation was not required for this study in accordance with the national legislation and the institutional requirements.

## Author contributions

ZZ, WL, and YZ: conceptualization. XF, YJ, and XY: methodology. ZZ, XF, YJ, and XY: software and project administration. ZZ: validation and formal analysis. LL and JC: investigation. XZ, YJ, and XY: resources. LL: data curation. ZZ, YJ, and XY: writing—original draft preparation. WL: writing—review and editing, visualization, and supervision. XF and YF: funding acquisition. All authors contributed to the article and approved the submitted version.

## Footnotes

1
https://tcga-data.nci.nih.gov

2
http://www.cgga.org.cn/

3
http://xenabrowser.net

4
http://www.zhounan.org/ferrdb/current/

5
https://wiki.cancerimagingarchive.net

6
https://www.cbica.upenn.edu/captk

## Supplementary material

Supplementary Figure 1 Flow chart.

Supplementary Figure 2 Venn diagram shows overlaps between genes conducted by univariate Cox regression analysis and DEGs.

Supplementary Figure 3 Based on the surv_cutpoint function in the survminer R package, the cut-off value of risk-score was determined to be −0.1.

Supplementary Figure 4 Distribution of risk-score **(top)**; survival time **(bottom)**.

Supplementary Figure 5 Summary ROC curves for the network (TC-mask and WT mask).
