# Supplementary figures and images for: Multiparametric magnetic resonance imaging-derived deep learning network to determine ferroptosis-related gene signatures in gliomas
Source: Front Neurosci. 2022 Dec 20;16:1082867. doi: 10.3389/fnins.2022.1082867 (PMC9808079; doi:10.3389/fnins.2022.1082867)

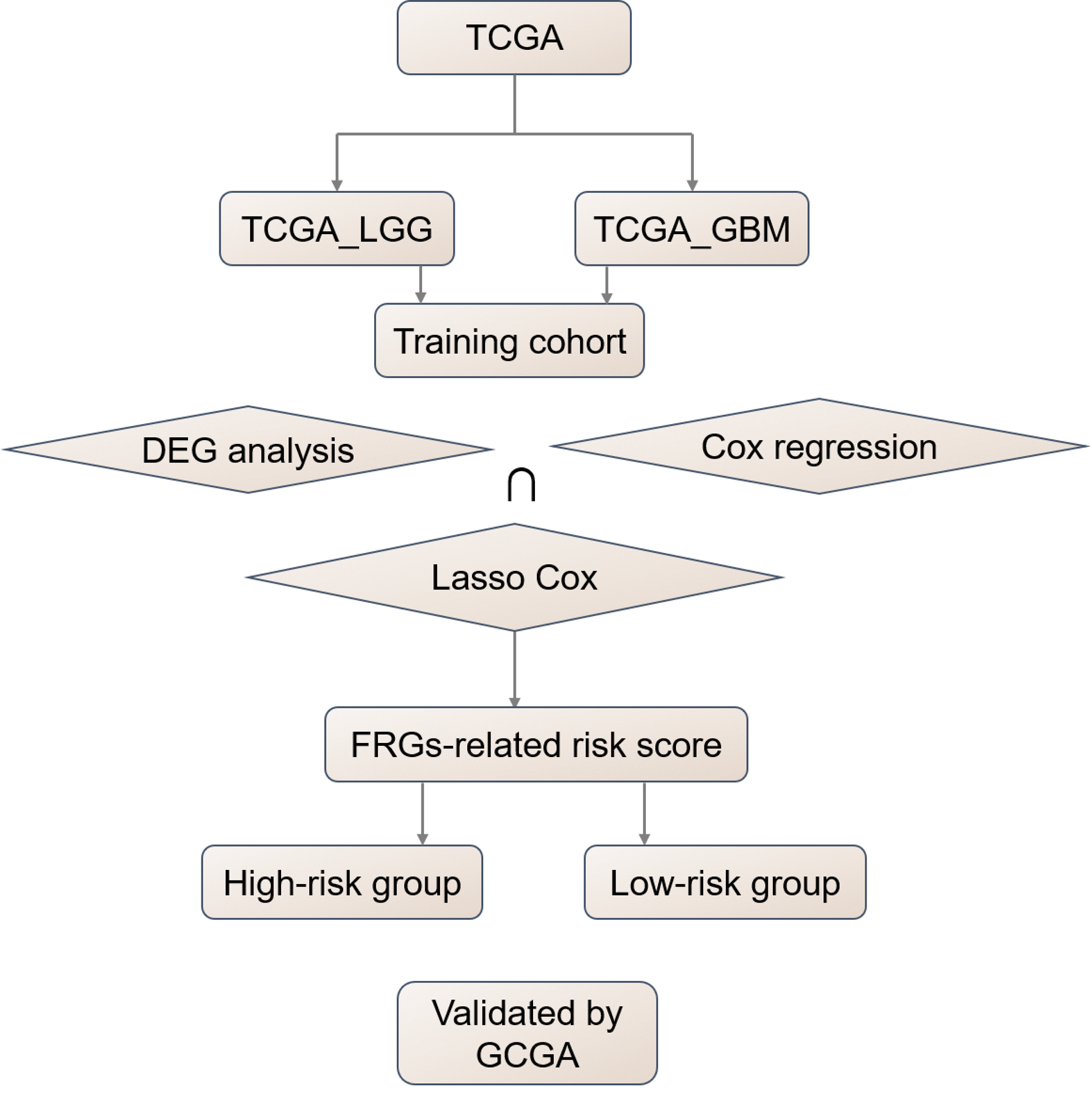

Supplement: Supplementary Figure 1 — Flow chart. [file Image_1.TIF]

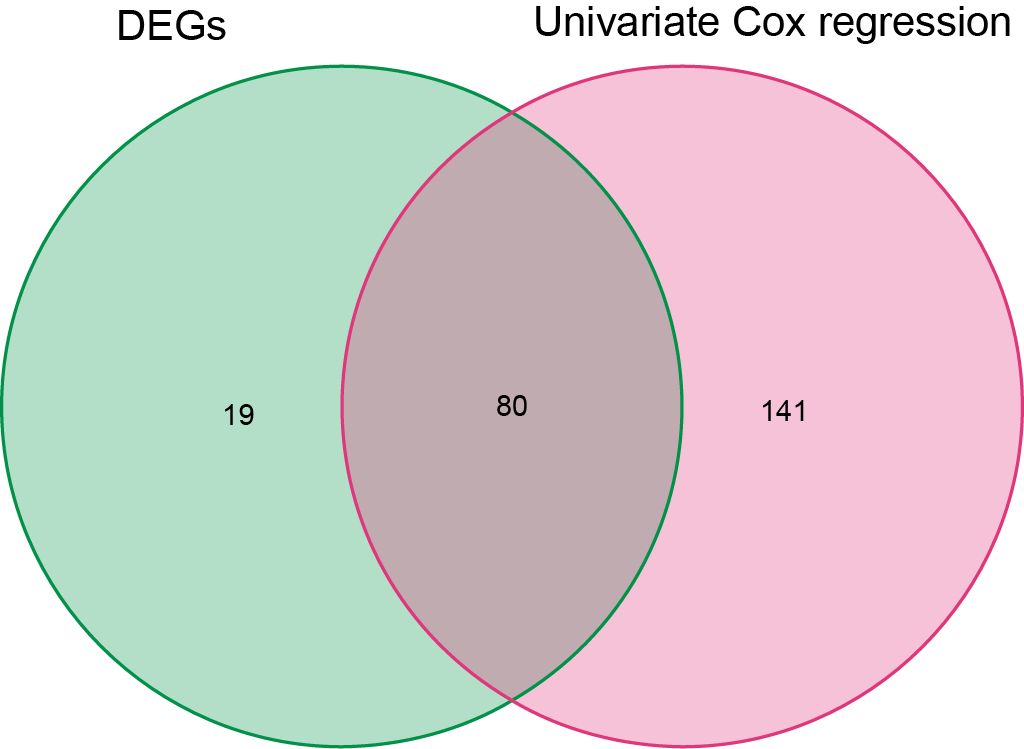

Supplement: Supplementary Figure 2 — Venn diagram shows overlaps between genes conducted by univariate Cox regression analysis and DEGs. [file Image_2.TIF]

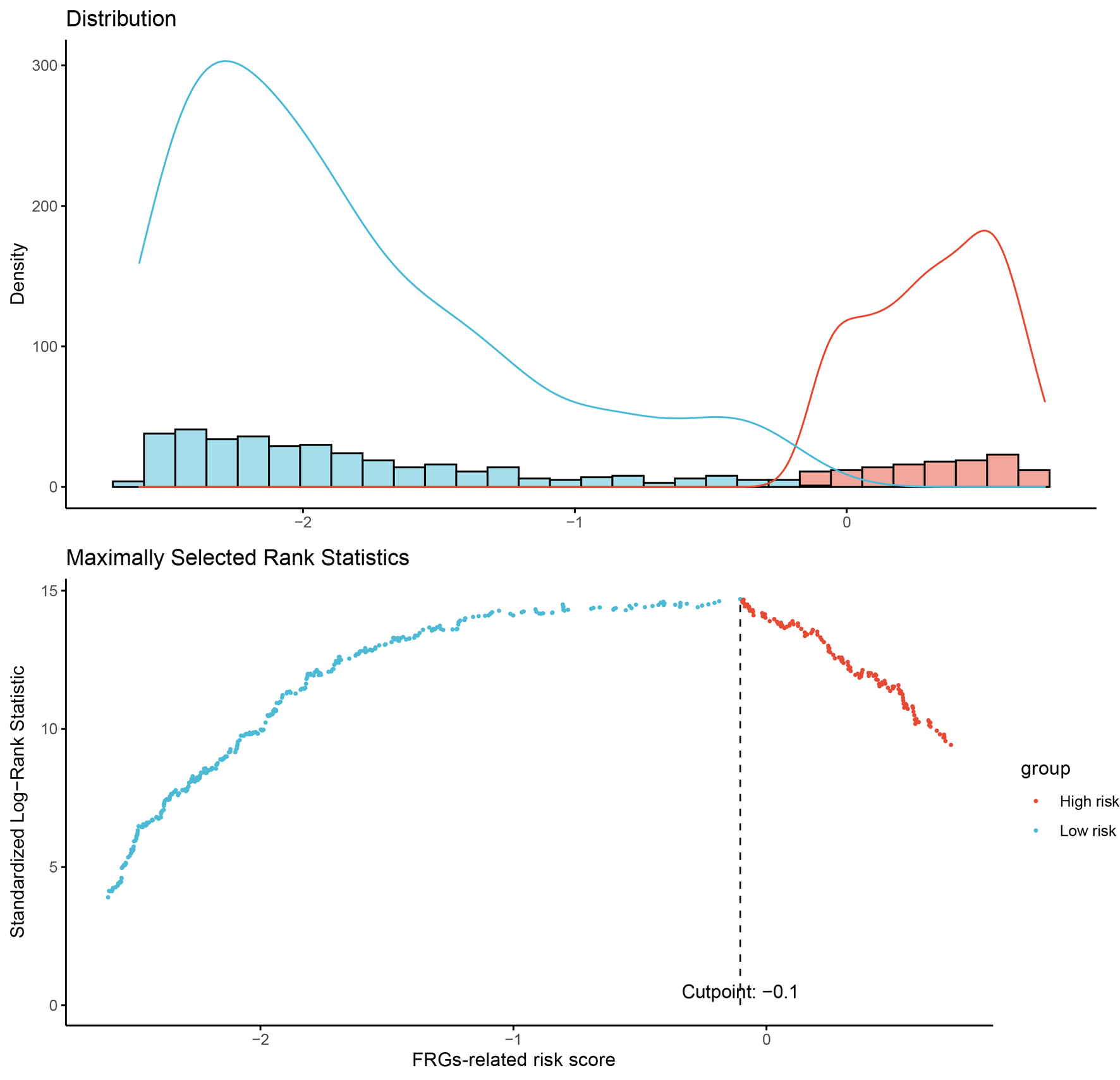

Supplement: Supplementary Figure 3 — Based on the surv_cutpoint function in the survminer R package, the cut-off value of risk-score was determined to be −0.1. [file Image_3.TIF]

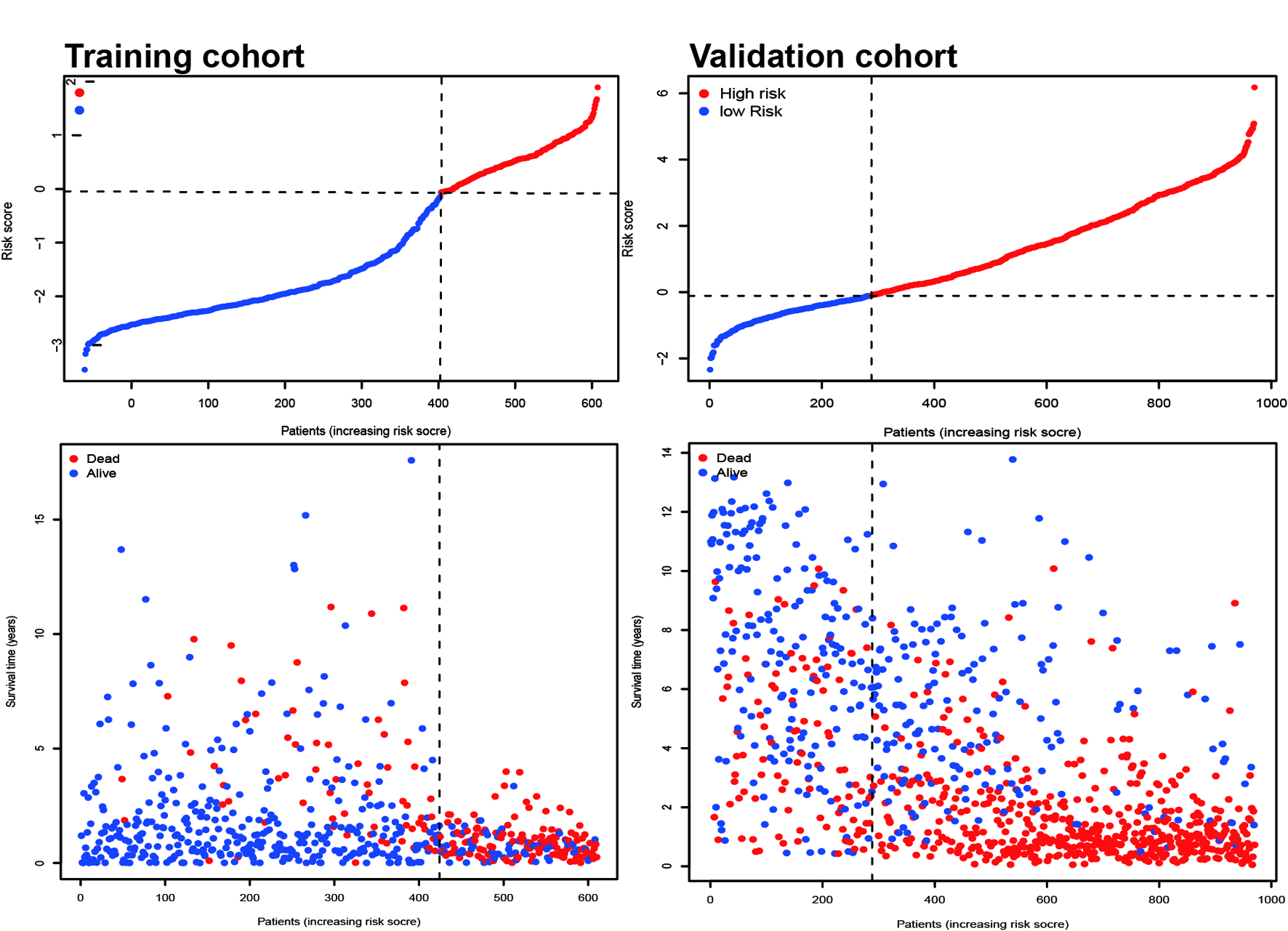

Supplement: Supplementary Figure 4 — Distribution of risk-score (top); survival time (bottom). [file Image_4.TIF]

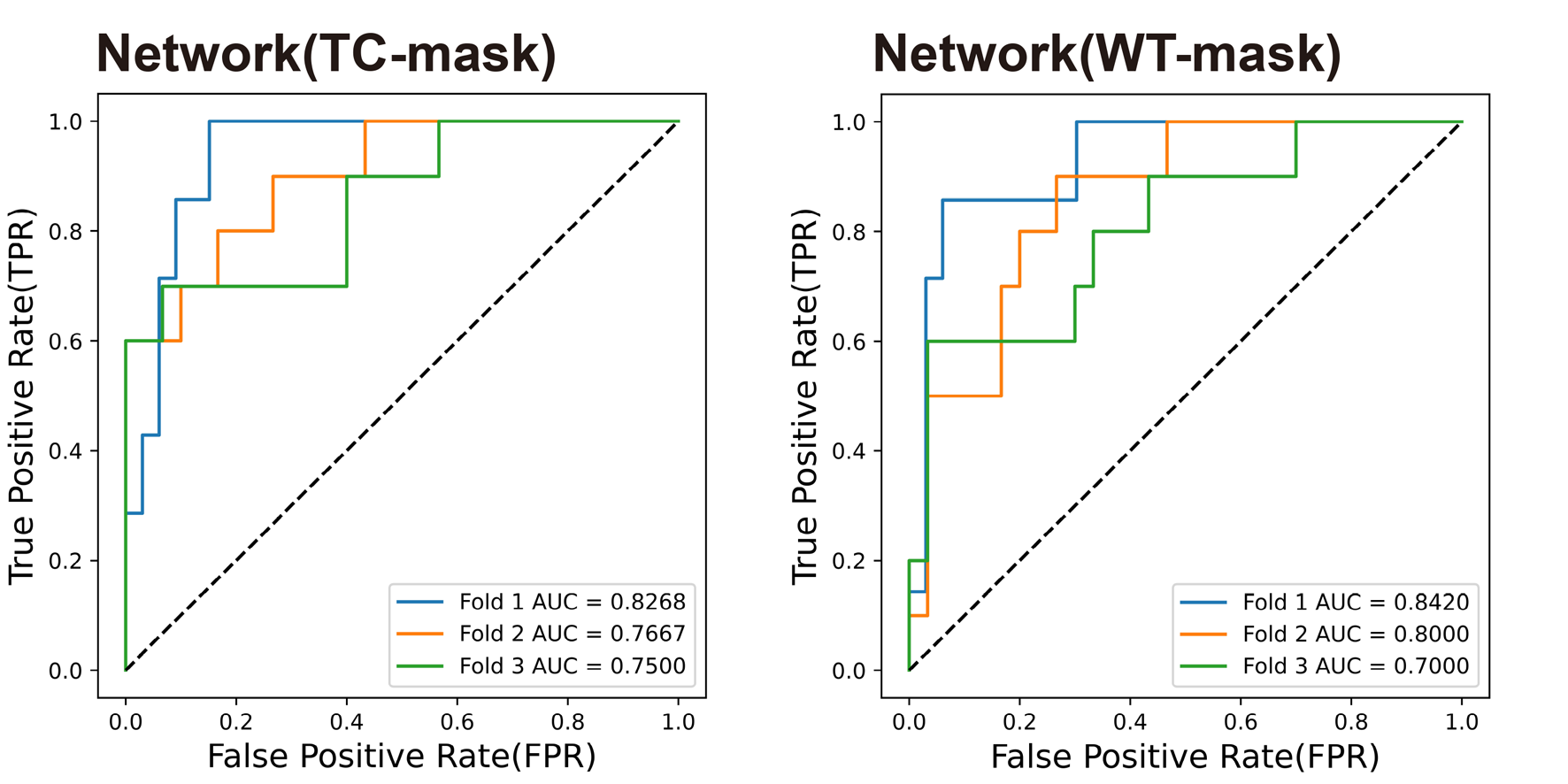

Supplement: Supplementary Figure 5 — Summary ROC curves for the network (TC-mask and WT mask). [file Image_5.TIF]
